# Environmental Conditions Affecting GABA Production in *Lactococcus lactis* NCDO 2118

**DOI:** 10.3390/microorganisms9010122

**Published:** 2021-01-07

**Authors:** Valérie Laroute, Roberto Mazzoli, Pascal Loubière, Enrica Pessione, Muriel Cocaign-Bousquet

**Affiliations:** 1 TBI, Université de Toulouse, CNRS, INRAE, INSA, 31077 Toulouse, France; loubiere@insa-toulouse.fr; 2 Department of Life Sciences and Systems Biology, University of Turin, Via Accademia Albertina 13, 10123 Turin, Italy; roberto.mazzoli@unito.it (R.M.); enrica.pessione@unito.it (E.P.)

**Keywords:** gamma-aminobutyric acid (GABA), osmotic stress, salts, polyols, chloride, nutraceuticals

## Abstract

GABA (γ-aminobutyric acid) production has been widely described as an adaptive response to abiotic stress, allowing bacteria to survive in harsh environments. This work aimed to clarify and understand the relationship between GABA production and bacterial growth conditions, with particular reference to osmolarity. For this purpose, *Lactococcus lactis* NCDO 2118, a GABA-producing strain, was grown in glucose-supplemented chemically defined medium containing 34 mM L-glutamic acid, and different concentrations of salts (chloride, sulfate or phosphate ions) or polyols (sorbitol, glycerol). Unexpectedly, our data demonstrated that GABA production was not directly related to osmolarity. Chloride ions were the most significant factor influencing GABA yield in response to acidic stress while sulfate ions did not enhance GABA production. We demonstrated that the addition of chloride ions increased the glutamic acid decarboxylase (GAD) synthesis and the expression of the *gad*BC genes. Finally, under fed-batch conditions in a complex medium supplemented with 0.3 M NaCl and after a pH shift to 4.6, *L. lactis* NCDO 2118 was able to produce up to 413 mM GABA from 441 mM L-glutamic acid after only 56 h of culture, revealing the potential of *L. lactis* strains for intensive production of this bioactive molecule.

## 1. Introduction

Lactic acid bacteria (LAB) have found applications in several manufacturing processes, where they are used as food starters [1], biocontrol agents [2], food thickeners [3] and probiotics [4]. A more recent and promising LAB application, is the production of functional food, in which they act as nutraceutical vectors (when employed in situ) or as cell factories for biosynthesizing molecules of interest for human health, called postbiotics [5]. The request for postbiotics in the global market is expected to grow in the coming years due to increased aging of the population [6].

One of the most appreciated compounds produced by LAB is γ-aminobutyric acid (GABA), the product of glutamate decarboxylation, known to modulate neurological disorders associated with diminished inhibitory activity of the central nervous system [7]. GABA is a very promising molecule that can affect mood (inducing a relaxed state), lower blood pressure [8] and induce gut smooth muscle relaxation (reducing excessive gut motility in some stress-induced colitis). For these reasons, GABA is used to control irritable bowel disease and mild hypertension [7].

As far as food is concerned, several GABA-containing products are currently available, especially in the Asian market including GABA rice [9], GABA soymilk [10], and GABA soya-yogurt (obtained by growing *Lactobacillus brevis* OPY-1 in the presence of germinated soybean extracts) [11]. Other nutraceutical preparations, such as GABA tea [12] and GABA chocolate [13] have been reported. Functionalized chocolate, containing 0.28% GABA, has been used to attenuate psychological stress and its action has been experimentally demonstrated by measuring the level of salivary cortisol [13].

As regards the fermentative production of GABA, several strategies have been proposed to obtain enhanced GABA yields from cheap substrates. Good amounts were obtained by growing *Lactobacillus buchneri* in corncob hydrolysate and xylose [14] or *Lactobacillus rhamnosus* in fermented adzuki bean milk [15]. *Lactobacillus brevis* strains isolated from quinoa sourdough [16] or kimchi [17] fermentation as well as *L. brevis* cultures fortified with divalent cations (Mg^2+^) and tween-80 [18] also display promising GABA yields. Co-cultures of *Corynebacterium glutamicum* and *Lactobacillus plantarum* [18], as well as entrapped [19] or immobilized [20] *L. brevis* cells were also described together with immobilized GAD enzyme [21] as a means to obtain economically sustainable GABA production.

So far, the highest performing GABA-producers have been reported among Lactobacilli, however, also Lactococci, Streptococci and Bifidobacteria can also synthesize significant amounts of GABA [22]. The selection of LAB strains bearing the genetic determinants for glutamate decarboxylation is of high value. Nevertheless, the presence of genes encoding the glutamate decarboxylation system (made up of a glutamate decarboxylase, GAD and of a Glu/GABA antiporter) may not be sufficient to support GABA production. Frequently, this route is regulated by several external conditions, at various levels, in bacteria [23]. The factors which are known to positively regulate the glutamate decarboxylation pathway include the presence of Glu (the precursor amino acid) [23], the stationary phase stress [24], acidic shock [25], salt stress [24], osmotic stress [26] and anaerobiosis [27].

*L. lactis* strains are frequently employed in industrial processes due to their lack of pathogenicity [28], metabolic efficiency [29], phage resistance [30] and bacteriocin production that guarantee them a good competitive survival [31]. In previous studies, we demonstrated that *Lactococcus lactis* NCDO 2118 can obtain GABA from glutamine opportunely converted into glutamate and that glutamate can act as an enhancer of GABA biosynthesis [23] and that GABA production is increased by arginine and malate supplementation [32]. In the present investigation, the natural GABA producing strain *L. lactis* NCDO 2118 was grown under different experimental conditions generating osmotic stress. Both ionic (salts) and non-ionic (polyols) stressors were tested with the double aim to: (i) elucidate further control mechanisms involved in such a complex and efficient pathway and (ii) to optimize conditions for GABA production in view of future application in the nutraceutical industry for GABA-enriched food production.

## 2. Materials and Methods 

### 2.1. Organism and Growth Conditions

*Lactococcus lactis* subsp. *lactis* NCDO 2118, a non-dairy strain isolated from peas was used throughout this study.

#### 2.1.1. Cultures in Tubes

Cultures were grown in chemically defined medium (CDM) [33,34], supplemented with glucose (20 g/L) and L-glutamic acid (34 mM) (precursor for GABA production) under anaerobic conditions, i.e., in N_2_ atmosphere, in butyl rubber-stoppered tubes at a temperature of 30 °C. The initial pH was 6.6. Depending on the experiments, different concentrations of additional compounds were added into the medium: sorbitol, glycerol, sodium chloride, ammonium chloride, potassium chloride, sodium sulfate, ammonium sulfate, potassium sulfate, di-sodium phosphate, di-ammonium phosphate or di-potassium phosphate. All the experiments were performed in duplicate. Inoculation was with cells from precultures harvested during the exponential phase and concentrated in order to obtain an initial optical density at 580 nm (OD_580_) of 0.05 in the tubes. Biomass production was estimated every 30 min directly in the tube without sampling during the first 6 h of culture by the measurement of OD_580_ with spectrophotometer (Milton Roy, Spectronic 301, Pont Saint Pierre, France). Samples (1 mL) were collected at 0, 6, 24, 48 and 72 h in order to measure the direct OD_580_ (Biochrom, Libra S11, Cambridge, England), pH (pH meter Metrohm 744, Villebon Sur Yvette, France), osmolarity and GABA concentration (see below for the methods).

#### 2.1.2. Cultures in Bioreactor

For studying the expression of *gad*B, *gad*C and *gad*R and the GAD activity according to chloride presence, bacterial cultures were performed in 2 L Biostat B plus bioreactor (Sartorius, Melsungen, Germany) filled with CDM, glucose (20 g/L), L-glutamic acid (34 mM) and with or without NaCl (0.3 M) at 30 °C. The pH was maintained at 6.6 by addition of 10 N KOH until the culture reached an OD_580_ of 1 in order to reach enough biomass for further analytical procedures and then pH was no longer regulated.

For optimization of GABA production, bacterial cultures were performed in a 2 L Biostat B plus bioreactor (Sartorius, Melsungen, Germany) filled with M17 medium (BD Difco), Yeast extract (10 g/L Biokar), glucose (45 g/L), L-glutamic acid (137 mM) and NaCl (0.3 M). As soon as the glutamate concentration was lower than 34 mM, about 152 mM of L-glutamic acid was added. Cultures were incubated at 30 °C. pH was maintained at 6.6 by KOH addition during the first 11 h and then pH was regulated at 4.6.

Bacterial growth was monitored by measurement of OD_580_ (Biochrom Libra S11, 1 Unit of absorbance is equivalent to 0.3 g/L). Samples were collected every 30 min for HPLC determination of GABA concentration in the growth medium. For GAD activity and RT-qPCR the culture volume equivalent to cell quantity of 150 mg and 6 mg, respectively, were taken at 5 and 7 h of growth.

### 2.2. GABA Determination

GABA concentration in culture supernatants was measured by a HPLC system (Agilent Technologies 1200 Series, Waldbronn, Germany) as previously described [32]. Briefly, proteins in the sample were precipitated by adding four volumes of methanol to one volume of sample followed by overnight incubation on ice. The mixture was centrifuged and the supernatant kept for amino acid analysis. The amino acids were automatically derivatized with OrthoPhtalic Aldehyde (OPA) and 9-fluorenylmethyl-chloroformiate (FMOC-C1). The derivatives were separated on Hypersil AA-ODS column (Agilent Technologies) at 40 °C by a linear gradient of acetate buffer (pH 7.2) with triethylamine (0.018%), tetrahydrofuran (0.3%) and acetonitrile. A diode array detector at 338 nm for OPA derivatives and 262 nm for FMOC derivatives was used.

### 2.3. Glutamate Decarboxylase (GAD) Activity

For GAD activity measurement, 150 mg cells were washed twice with 0.2% KCl (m/v) and suspended in sodium acetate buffer (100 mM, pH 4.6) containing 4.5 mM MgCl_2_, 22% (*v*/*v*) glycerol and 1.5 mM DTT. Cells were disrupted by sonication and kept on ice during the treatment. Cell debris were removed by centrifugation for 15 min at 10,000× *g* at 4 °C. The supernatant was used for enzyme assays, and the protein concentration of the extract was determined by the Bradford method. Enzyme assay was realized with 0.5 mL of substrate solution, consisting of 20 mM sodium glutamate, 2 mM pyridoxal phosphate (PLP) incubated at 30 °C then mixed with 0.5 mL supernatant. Every 30 min until 4 h, 100 µL were sampled and inactivated by boiling for 5 min to stop the decarboxylation reaction. Reaction mixtures were subsequently analyzed for the presence of GABA using HPLC. One unit of enzyme activity was defined as the amount of enzyme which converted 1 nmol of glutamate per min and per mg of protein.

### 2.4. RNA Extraction and Gene Expression Analysis

A culture volume corresponding to 6 mg dry weight biomass was harvested and frozen immediately in liquid nitrogen. Before cell lysis, each sample was centrifuged (4 °C, 5 min, 4800 rpm), washed with 1 mL of TE buffer (Tris-HCl 10 mM, pH 8, EDTA 1 mM) and resuspended in 500 µL of TE buffer. Cells were disrupted at 4 °C (6.5 m/s, 4 cycles, 30 s, 1 min cooling on ice) on a minibead beater (Biospec Products) with glass beads (0.6 g), 25 µL of SDS (20%), and 500 µL of phenol (pH 4.7). Cell debris and phenol were eliminated by centrifugation and (4 °C, 25 min, 13,000 rpm). RNA from the aqueous phase was extracted with RNeasy midi kit (Qiagen). The standard protocol (precipitation, washing and elution) including the DNase I treatment described in the manufacturer’s instructions. RNA concentration and quality were measured using a NanoDrop spectrophotometer and an Agilent Bioanalyzer (Santa Clara, CA, USA). The samples were subjected to reverse transcription using Super Script II reverse transcriptase (LifeTechnology), as previous described [35]. RT-qPCR was performed using a SYBR green-based detection protocol (Life Technology) with an Opticon 2 real-time PCR detection system (Bio-Rad) and MyIQ software (Bio-Rad). Primers (Table 1) specificity and PCR efficiency were analyzed on genomic DNA range prior to quantification. The *tuf* gene was used as an internal standard for normalization. Variations of gene expression between conditions were calculated with the ΔΔCt method [36] and expressed as fold changes.

### 2.5. Osmolarity Measurement

Osmolarity was measured in the samples with a freezing point osmometer (Roebling Osmometer automatic, Berlin, Germany). The instrument was calibrated using 300 and 900 mOsm/kg standard solutions.

### 2.6. Growth Rate Estimations

During the exponential growth phase, the specific growth rate was maximum and constant. The maximum growth rate (*µ*_max_) was calculated from five consecutive OD_580_ measurements during cultures in tubes between 0.5 to 2.5 h according to the following formula: (*µ*_max_ = ∆lnOD_580_/∆t, where t is time). For the instantaneous growth rate (*µ*) measured throughout the growth of *L. lactis* NCDO 2118 into the bioreactor, the following formula was used: *µ* = (lnX_n + 1_ − lnX_n_)/(t_n + 1_ − t_n_), where X was biomass and t is time).

### 2.7. Statistical Treatments

To analyze the linear correlation between two variables (GABA vs. osmolarity or *µ*_max_ vs. osmolarity), the correlation coefficient was calculated with the correlation Pearson test.

## 3. Results

### 3.1. Impact of Osmolarity on the Growth Rate

**Figure 1:**
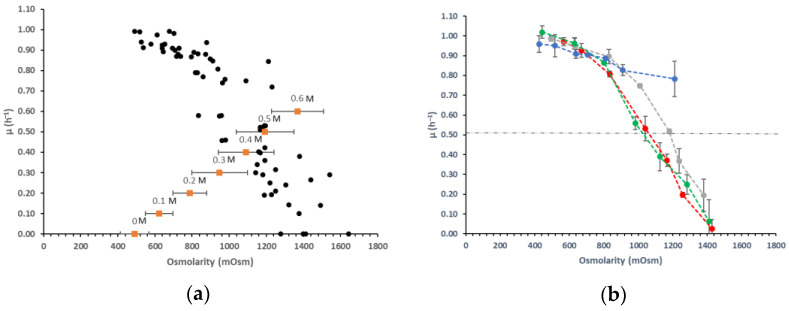
(**a**) Specific growth rate (● h^−1^) of *L. lactis* NCDO 2118 measured in chemically defined medium (CDM) containing various concentrations of salts or polyols (0 to 0.6 M) and corresponding range of osmolarity at a given salt or polyol molarity (■ and the given concentration above in M) after 6 h of growth (**b**) mean of specific growth rate (h^−1^) of *L. lactis* NCDO 2118 measured in CDM containing various concentrations of chloride ●, phosphate ●, or sulfate ● salts or polyols ●.

Cultures of *L. lactis* NCDO 2118 were performed in duplicates in glucose–glutamate–CDM medium supplemented with various salts or polyols (two polyols and nine salts) at different concentrations ranging from 0 to 0.6 M. Specific growth rates and osmolarity were both determined in each condition and presented in Figure 1. The osmotic pressure developed in the media ranged from 490 (0 M) to 1644 mOsm (0.6 M) (Figure 1a). Compared to these variations, the osmolarity changes between each tested salt or polyol at a fixed molarity were weak, as well as along the culture for all assays (± 46 to 201 mOsm) (supplementary data Table S1). Whatever the salt or polyol added in the medium, the measured osmolarity was thus well related with its molarity throughout the growth of *L. lactis*. Experimental results showed a negative correlation between growth rate and osmolarity (Figure 1a) (correlation of −0.89) indicating that growth is significantly affected by increasing osmolarity. In order to better understand this effect, we analyzed the variation of the growth rate as a function of the ions (chloride, sulfate, phosphate) independently of the counterions (sodium, ammonium, potassium) (Figure 1b). Whatever the counterion used for a given ion, the results were similar as well as for the two polyols with a clear negative effect of the osmolarity on the growth rate. Each value of *µ*_max_ corresponded here to the mean of growth rates for a given ion or polyol. The maximal growth rate obtained in non-supplemented medium was 1.02 h^−1^. If we look closer at the results, some variations between the different ions can be noticed. In the presence of polyols or phosphate ions, the growth rates did not decrease too much, until an osmolarity of 1100 mOsm. For chloride or sulfate ions, the levels of growth rate dropped rapidly from 800 mOsm. The inhibition effect of each salt or polyol was estimated quantitatively by the value of osmolarity causing a reduction by half of the maximal growth rate (Ki = value of osmolarity when the growth rate is 0.51 h^−1^). These inhibition constants were similar for chloride or sulfate ions (1050 mOsm) and higher for phosphate ions (1250 mOsm), while the Ki was not reached with polyols. The growth rate indeed declined to only 22% at 1250 mOsm with polyols.

### 3.2. GABA Production under Osmotic Stress

**Figure 2:**
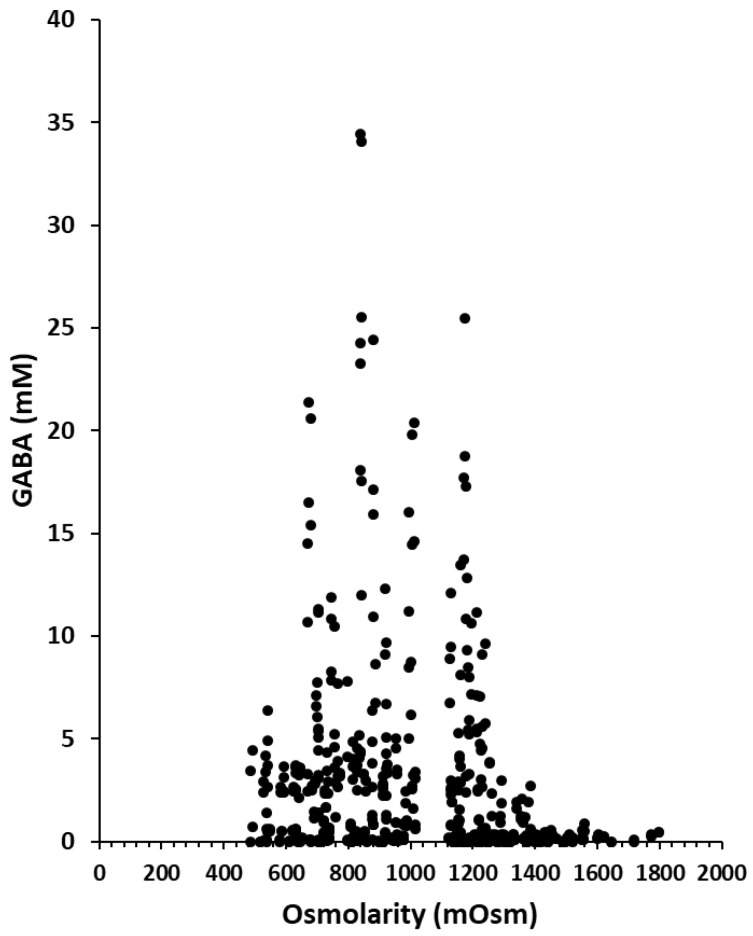
γ-aminobutyric acid (GABA) production (mM) in CDM containing various concentrations of chloride, phosphate or sulfate salts or polyols during growth of *L. lactis* NCDO 2118 at different time of the culture (6, 24, 48 and 72 h).

GABA production by *L. lactis* NCDO 2118 was investigated in the different osmotic stress conditions described above. The GABA concentrations produced over time for each sampling time and each level of concentration of added salt or polyol were plotted as a function of the measured osmolarity for each of these samples (Figure 2). GABA production varied significantly between 0 and 34 mM and was not correlated to osmolarity (as shown by the dot cloud on the Figure 2 and by the low value of data correlation estimated at −0.19). In other words, GABA concentration changed significantly at a fixed osmolarity with the largest variation at 830–860 mOsm, from a minimal production of 0.09 mM to a maximal production of 34 mM. For a fixed osmolarity, effects on GABA production were thus very different. We also verified that in all the different conditions analyzed separately, the lack of correlation was also obtained in most cases (supplementary data Figure S1).

**Figure 3:**
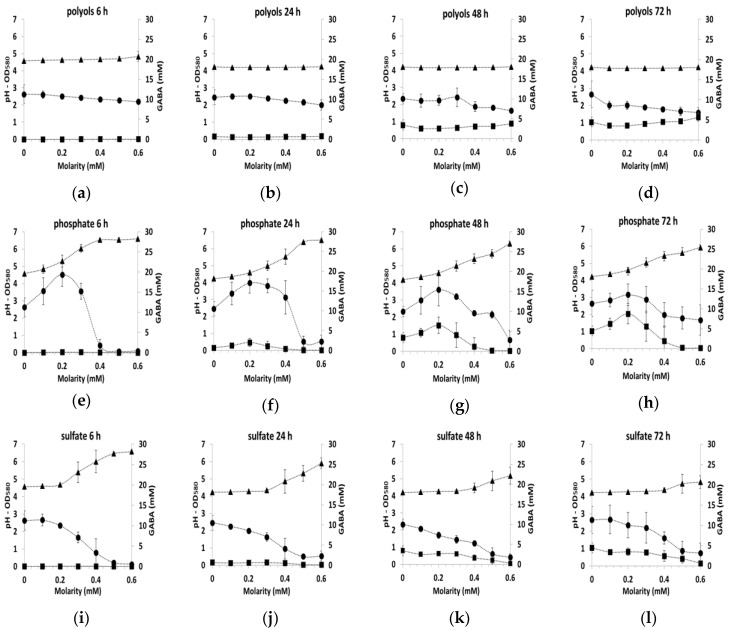

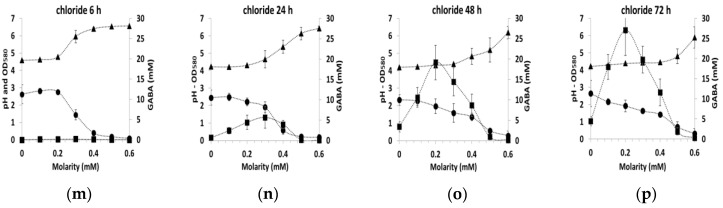
GABA production (mM, ■), pH (▲) and biomass production (OD_580_, ●) in CDM containing various concentrations of polyols (means of both polyol) (**a**) 6 h, (**b**) 24 h, (**c**) 48 h, (**d**) 72 h, or various concentrations of salts (means of counter ions) (**e**) phosphate 6 h, (**f**) phosphate 24 h, (**g**) phosphate 48 h, (**h**) phosphate 72 h (**i**) sulfate 6 h, (**j**) sulfate 24 h, (**k**) sulfate 48 h, (**l**) sulfate 72 h (**m**) chloride 6h, (**n**) chloride 24 h, (**o**) chloride 48 h, (**p**) chloride 72 h during the growth of *L. lactis* NCDO 2118.

In order to gain insights into these important differences in GABA production, we analyzed more closely the results as a function of time of culture for each added molecule and joined to the analysis other culture indicators such as pH and biomass concentration data (Figure 3). Since the results with the different counterions (sodium, ammonium and potassium) were similar for each ion (chloride, phosphate, sulfate), data were averaged independently of the counter ion at each time point (6, 24, 48 or 72 h of the culture) and standard deviations were given on the plot. Similarly, the mean value of both polyols (sorbitol and glycerol) was calculated.

With polyols, the level of biomass was relatively constant irrespective of the polyol concentration in the medium or the sampling time and the pH remained also constant in the range 4.1–4.2. (Figure 3a–d). GABA production was observed only at 48 and 72 h and reached 2.5 to 5 mM. With ions, more variable profiles were observed as function of the sampling time (Figure 3e–p). Growth was generally inhibited by salts for concentrations higher than 0.2–0.3 mM, as indicated by the decrease in biomass production associated with the increase in pH above 4.1–4.2. For the phosphate ion only, an increase in growth was detected before the growth inhibition (between 0 to 0.2–0.3 M from OD 2.7 to 3.6). This was probably related to the buffering capacity of phosphate ions.

In all the tested conditions, GABA production occurred only in the acidic environment, namely when pH was between 4.3–5.0. In the presence of sulfate, the maximum GABA production was about 2.5 to 3.2 mM, while with phosphate salts, this production reached 4.4 to 8.7 mM. This difference in the production was at least partially explained by the higher biomass production in the presence of phosphate salts. While the pH and biomass production profiles with chlorides were very similar to those with sulfate, GABA production diverged greatly. With chlorides, GABA production reached 6 mM at 24 h and exceeded 30 mM at 72 h. This early and efficient production was the maximum observed here, indicating that GABA production is activated by chlorides.

In order to better understand the influence of chlorides on the GABA production, we compared the activity of glutamate decarboxylase (GAD) and the expression of the *gad* genes in CDM with or without 0.3 M NaCl in a bioreactor (Table 2). The chloride addition provoked a strong increase in the GAD synthesis from 1 to 37 mmole/min/g on average. This was accompanied by a mean of 15-fold expression increase for each gene of the *gadCB* operon while the expression of the *gadR* regulator remained constant.

### 3.3. GABA Production in Bioreactor

**Figure 4:**
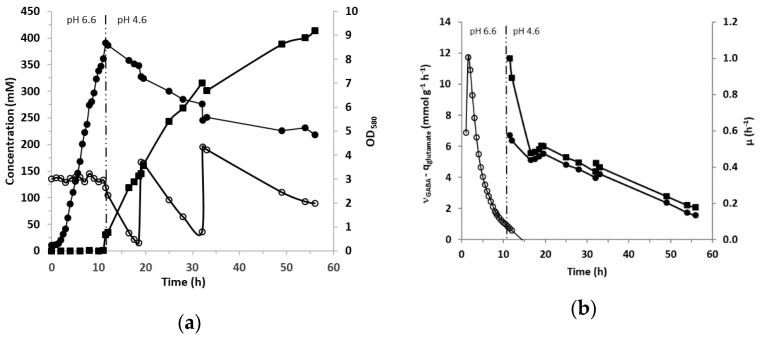
(**a**) Evolution of GABA production (mM; ■), glutamate concentration (mM; ○) and biomass production (OD_580_, ●) during growth of *L. lactis* NCDO 2118 in M17 containing YE (10 g/L), glucose (45 g/L), NaCl (0.3 M) and 137 mM of initial GABA concentration. pH was regulated to 6.6 until 11 h of culture then shifted to 4.6 and maintained at this value for the remainder of the fermentation. Two additions of approximately 152 mM glutamate were added at 19 and 32 h of culture. (**b**) Evolution of specific growth rate (h^−1^; ○), specific glutamate consumption (mmol g^−1^ h^−1^; ●) and specific GABA production (mmol g^−1^ h^−1^; ■) during the 56 h of culture. Negative growth rates associated to the biomass decrease after 15h are not represented.

Previous experiments were performed in tubes with a synthetic medium (CDM) that is exclusively used in the laboratory. Hence, the potential of *L. lactis* NCDO 2118 to produce GABA in more realistic conditions was tested with a rich medium favoring growth (M17 medium supplemented with yeast extract (10 g/L) and glucose (45 g/L)) and in controlled conditions in a bioreactor with an initial osmolarity of 983 mOsm. NaCl (0.3 M) was also added into the medium since the tests in tubes described above demonstrated that it is the most important factor promoting GABA production. The initial L-glutamic acid concentration was 137 mM, then, two additions of about 151 mM each were made when the concentration dropped to less than 10–20 mM. pH was maintained at 6.6 until 11 h to obtain high biomass production before changing to an acidic pH of 4.6–4.7 which corresponded to the optimal pH for the GAD enzyme [37]. This complex medium associated with maintaining pH at 6.6 made it possible to achieve a biomass of 2.52 g/L (OD_580_ = 8.4) (Figure 4a). The biomass was thus increased almost by a factor of three compared to the conditions in tubes (about OD_580_ = 3 without salts or polyols). As soon as the pH was regulated to 4.6, GABA production started, while the growth instantly stopped. Then the biomass level slowly decreased all along the GABA production phase, suggesting the arrest of cell multiplication. Within 7 h the cells converted 90% of the supplied glutamate into GABA (Figure 4a). The first glutamate addition (about 136 mM) was consumed at 78% in 13 h. After the second glutamate addition, a final GABA concentration as high as 413 mM was reached after 56 h of culture. The profiles of the specific rates of glutamate consumption and GABA production are shown in Figure 4b. They followed the same evolution and decreased slowly while the specific growth rate was zero after 11 h of growth (Figure 4b).

## 4. Discussion

Some specific lactic acid bacteria (LAB) strains produce bioactive molecules such as GABA, the product of glutamate decarboxylation, by the glutamic acid decarboxylase (GAD) enzyme. The biosynthesis of GABA by microorganisms has been described as a response to abiotic stresses, such as acidity and starvation [38], with different behaviors depending on the species considered. Additionally, osmotic stress can trigger GABA production [26,39,40,41]. Several osmoregulatory solutes [42,43,44] have been described in the literature, however, the role of GABA in osmotic stress tolerance has not been clearly established yet. Here, we investigated the relationship between medium osmolarity and GABA production in *L. lactis* NCDO 2118, a native GABA producer [23].

The data obtained in this work show that, at osmolarities ranging between 487 to 1795 mOsm, ionic (salts) and non-ionic (polyols) stresses had different effects on growth and GABA production. Hyperosmotic conditions generated by polyols were much less harmful for growth than those linked to salts at similar osmolarities. Quite similar observations have been made for *Lactobacillus plantarum* [45] under sugar stress. Polyols have been described as osmoprotective compounds [46,47,48]. Although polyols did not negatively affect the growth rate, the related increased osmolarity did not result in a sharp GABA increase. No clear correlation between GABA synthesis and osmolarity was observed in the present study, considering all the different osmoregulatory solutes tested. This indicated that the relationship between osmolarity and GABA production was more complex than previously hypothesized and this was at least partially explained by variable influence of osmolarity solutes on growth performances, which also directly impact GABA production.

The primary parameter affecting GABA synthesis was confirmed here to be the pH: actually, GABA production is detectable only under acidic conditions. LAB metabolism is mainly directed to the production of organic acids such as lactic and acetic acid. This implies that they have to frequently face acid stress and, therefore, they have developed different mechanisms to mediate acid resistance, such as the GAD pathway [49]. In our study, the activation of this pathway occurred after the pH shifted to 4.6 and during the stationary phase as already shown in previous studies.

Curiously, GABA production was very heterogeneous depending on the nature of added salts (sodium chloride, ammonium chloride, potassium chloride, sodium sulfate, ammonium sulfate, potassium sulfate, di-sodium phosphate, di-ammonium phosphate or di-potassium phosphate). The differences observed did not relate to the NH_4_^+^, K^+^ and Na^+^ cations used. Other cations have been described as modulators of GABA production since they act as activators (Ca^2+^ and Mg^2+^) [50] or inhibitors (Cu^2+^, Fe^2+^, Fe^3+^, Ag^+^) of the GAD enzyme [51]. The sulfate ion was not able to enhance the GAD activity of *L. lactis* NCDO2118 although it was previously identified as an activator of GAD in another LAB [52]. Conversely, we confirmed that chloride anions trigger an increase in GABA production. Actually, in the presence of chloride ions, the GABA concentration was enhanced by a factor of four to 10, thus leading to an approximate 90% conversion of glutamate at 72 h (Figure 3p). Although GABA biosynthesis did not correlate with osmolarity, the chloride ion concentration seems to play an important role, supporting the idea of its industrial use to promote GABA-production optimization. Our results for the expression of the *gadCB* and *gadR* genes confirmed the mechanism of activation of the *gadCB* promoter P*gad* by chloride, demonstrated previously with reporter genes [53] as well as the constitutive expression of the regulator *gadR*. In addition we demonstrated here that this gene activation provoked a profound change of GAD protein synthesis with an increased factor of 37 in the presence of chloride, which is even higher than the fold change of 15 observed for the expression of the corresponding gene *gadB*. We noted that the higher the concentration of chloride, the higher the production of GABA, within the limit of the strain growth pattern. Indeed, cell yield was reduced by the inhibition of growth at a too high chloride concentration and consequently GABA production decreased.

In this work, we also investigated the potential of GABA production of *L. lactis* NCDO 2118 in a bioreactor. Growth was favored by the choice of a suitable medium and an adapted pH regulation strategy (pH was maintained at 6.6 before shifting to 4.6 to induce activation of GAD activity). In these conditions, after two additions of 152 mM glutamate, *L. lactis* NCDO 2118 produced high GABA levels, reaching 413 mM (42.6 g/L) in 56 h. It has to be underlined that this yield is the highest obtained so far by using this strain. In a previous piece of work, only a production of 8.6 mM was obtained for *L. lactis* NCDO 2118 [32]. A number of reviews [54,55,56,57] focussing on GABA production have reported that LAB, particularly *Lactobacillus* strains, are among the best producers. However, a study on eight *Lactobacillus brevis* strains [58] reported a GABA titer ranging between 16 and 258 mM after 72 h of culture (in presence of 267 mM glutamate), while *Lactobacillus plantarum* (nine strains examined) produced only between 18 and 51 mM GABA. Under optimized conditions and for a particular *Lactobacillus* strain (*Lactobacillus brevis* NCL912), a massive supply of glutamate could also enhance GABA production until 1005 mM [59]. It is thus clear that GABA production strongly depends not only on environmental parameters but on the bacterial strains/species as well.

Concerning *Lactococcus* strains, only a few studies on GABA production are available in the literature and most of them were focused on *L. lactis* NCDO 2118. However, other strains were also identified as GABA producers such as *Lactoccocus lactis* 01-7 (isolated from food starters) [60] or *Lactoccocus lactis* B (isolated from kimchi) [61]. Similarly, to what was observed for lactobacilli, lactococci are therefore expected to exhibit great diversity concerning GABA production.

## 5. Conclusions

The results presented here have provided additional perspectives to previous research demonstrating the high potential of *L. lactis* NCDO 2118 to produce GABA. In addition to glutamate and glutamine [23], malate and arginine [32], and also chloride can be used to enhance the industrial production of GABA, a molecule useful to treat several neurological, psychiatric, and cardiovascular diseases, whose global market is expected to increase in the coming years. Taken together, these results provide news insights for the use of the *Lactococcus* strains for GABA production and open the way for the exploration of GABA production in this, as yet, under-exploited bacterial niche.

## Figures and Tables

**Table 1:** Sequences of primers used for quantitative RT-PCR.

Gene Name	Forward Primer	Reverse Primer
*gadB*	CAACATGATCGCTGACCTTTGG	GCCATTCCACCAAGCATACAAG
*gadC*	CAGCAGAAATGGCGACGGTTG	GCTCCCCTAAAGTTTGGCTCAC
*gadR*	ATGCAAGTGCCATTAGCTGAGTAC	AGTCCCAAGCTTCGTTTTAACGG
*tuf*	AAGGAGTGGTTTGTCAGTGTCG	CTTGGTGCTTTGAACGGTGAAC

**Table 2:** GAD activities and gene expression fold changes (FC) for *gad*B, *gad*C and *gad*R in glucose-glutamate-CDM supplemented with or without NaCl (0.3 M) after 5 and 7 h of culture in bioreactor. (FC mean with n = 3, GAD activities mean with n = 2).

	CDM	CDM + NaCl	Gene Expression FC by NaCl Addition		
Time	GAD Activity	GAD Activity			
(h)	mmole/min/g	mmole/min/g	***gad*B**	***gad*C**	***gad*R**
**5**	1.8 ± 0.4	36.4 ± 9.0	10.7 ± 3.1	13.6 ± 4.9	0.6 ± 0.3
**7**	2.3 ± 1.0	37.0 ± 2.0	17.4 ± 4.2	17.3 ± 3.0	1.4 ± 0.3

## Data Availability

Not applicable.

## Supplementary Materials

The following are available online at https://www.mdpi.com/2076-2607/9/1/122/s1, Table S1: Osmolarity measures in glucose-glutamate-CDM supplemented with various salts or polyols at different concentrations ranging from 0 to 0.6 M after 6, 24, 48 or 72 h of culture. (mean with n = 22), Figure S1: GABA production (mM) in CDM containing various concentrations of salts or polyols during growth of *L. lactis* subsp. *lactis* NCDO 2118 at different time of the culture (6, 24, 48 and 72 h).
