# Supplementary material for: Environmental Conditions Affecting GABA Production in Lactococcus lactis NCDO 2118
Source: Microorganisms. 2021 Jan 7;9(1):122. doi: 10.3390/microorganisms9010122 (PMC7825684; doi:10.3390/microorganisms9010122)
Supplement: Supplementary file 1 [file microorganisms-09-00122-s001.pdf]

Table S1. Osmolarity measures in glucose-glutamate-CDM supplemented with various salts or polyols at different concentrations ranging from 0 to 0.6 M after 6, 24, 48 or 72 h of culture. (mean with n=22)

**Molarity**	**Osmolarity** (mOsm ± SD)			
(mM)	6 h	24 h	48 h	72 h
0	490 ± 77	495 ± 74	492 ± 48	513 ± 46
0.1	623 ± 73	636 ± 73	644 ± 74	664 ± 69
0.2	788 ± 91	797 ± 104	808 ± 96	840 ± 99
0.3	948 ± 150	980 ± 150	1017 ± 168	1042 ± 171
0.4	1091 ± 151	1126 ± 162	1129 ± 171	1163 ± 165
0.5	1193 ± 155	1250 ± 180	1282 ± 200	1301 ± 201
0.6	1368 ± 141	1397 ± 156	1439 ± 171	1493 ± 176

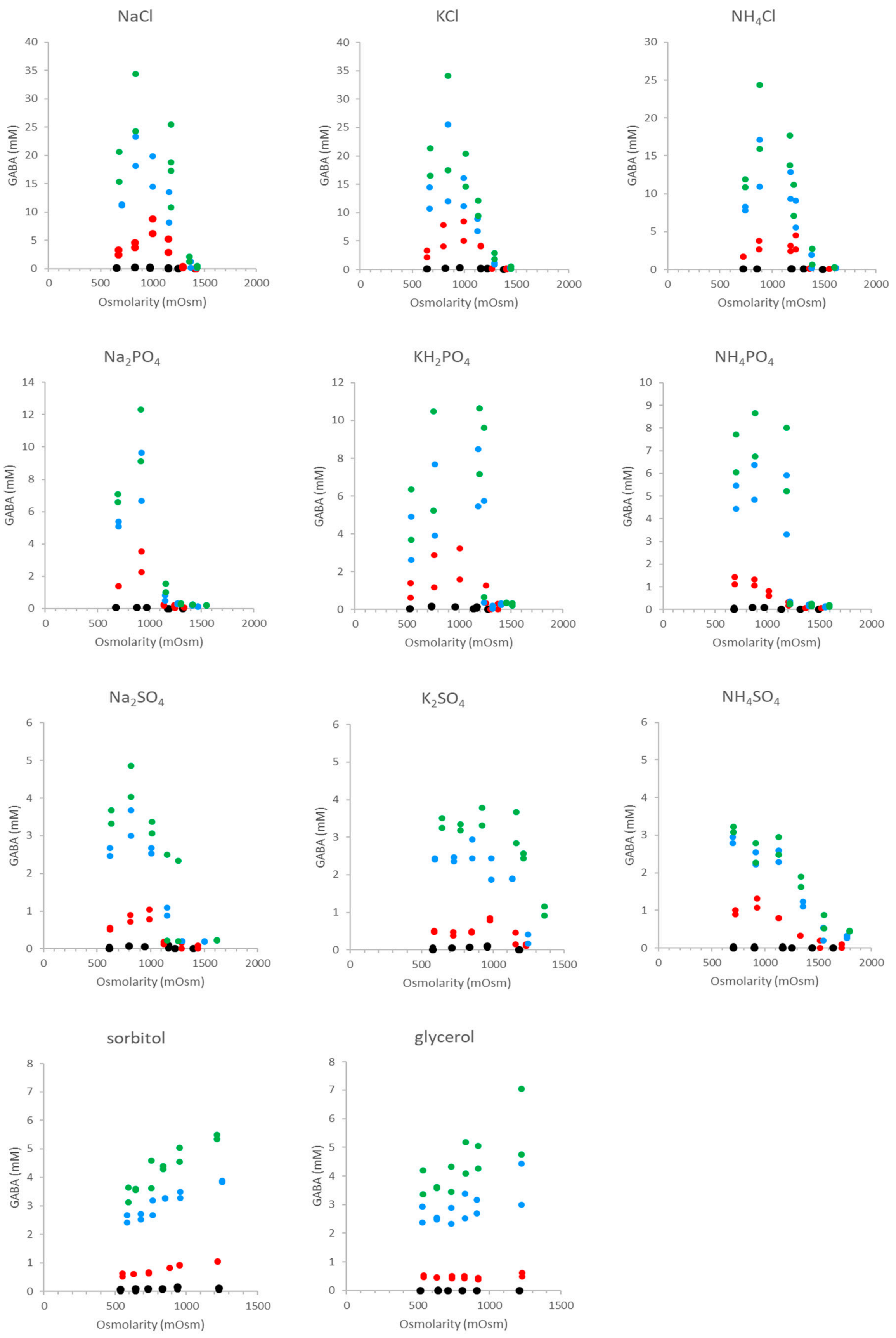

Figure S1. GABA production (mM) in CDM containing various concentrations of salts or polyols during growth of *L. lactis* subsp. *lactis* NCDO 2118 at different time of the culture (6 ●, 24 ●, 48 ●and 72 ●hours).
